# Cost-Effectiveness of 2009 Pandemic Influenza A(H1N1) Vaccination in the United States

**DOI:** 10.1371/journal.pone.0022308

**Published:** 2011-07-29

**Authors:** Lisa A. Prosser, Tara A. Lavelle, Anthony E. Fiore, Carolyn B. Bridges, Carrie Reed, Seema Jain, Kelly M. Dunham, Martin I. Meltzer

**Affiliations:** 1 Child Health Evaluation and Research Unit, Division of General Pediatrics, University of Michigan Health System, Ann Arbor, Michigan, United States of America; 2 Ph.D. Program in Health Policy, Harvard University, Cambridge, Massachusetts, United States of America; 3 Influenza Division, National Center for Immunization and Respiratory Diseases, Centers for Disease Control and Prevention, Atlanta, Georgia, United States of America; University of Hong Kong, Hong Kong

## Abstract

**Background:**

Pandemic influenza A(H1N1) (pH1N1) was first identified in North America in April 2009. Vaccination against pH1N1 commenced in the U.S. in October 2009 and continued through January 2010. The objective of this study was to evaluate the cost-effectiveness of pH1N1 vaccination.

**Methodology:**

A computer simulation model was developed to predict costs and health outcomes for a pH1N1 vaccination program using inactivated vaccine compared to no vaccination. Probabilities, costs and quality-of-life weights were derived from emerging primary data on pH1N1 infections in the US, published and unpublished data for seasonal and pH1N1 illnesses, supplemented by expert opinion. The modeled target population included hypothetical cohorts of persons aged 6 months and older stratified by age and risk. The analysis used a one-year time horizon for most endpoints but also includes longer-term costs and consequences of long-term sequelae deaths. A societal perspective was used. Indirect effects (i.e., herd effects) were not included in the primary analysis. The main endpoint was the incremental cost-effectiveness ratio in dollars per quality-adjusted life year (QALY) gained. Sensitivity analyses were conducted.

**Results:**

For vaccination initiated prior to the outbreak, pH1N1 vaccination was cost-saving for persons 6 months to 64 years under many assumptions. For those without high risk conditions, incremental cost-effectiveness ratios ranged from $8,000–$52,000/QALY depending on age and risk status. Results were sensitive to the number of vaccine doses needed, costs of vaccination, illness rates, and timing of vaccine delivery.

**Conclusions:**

Vaccination for pH1N1 for children and working-age adults is cost-effective compared to other preventive health interventions under a wide range of scenarios. The economic evidence was consistent with target recommendations that were in place for pH1N1 vaccination. We also found that the delays in vaccine availability had a substantial impact on the cost-effectiveness of vaccination.

## Introduction

2009 pandemic influenza (A)H1N1 (pH1N1)was first identified in Spring 2009 and has continued to circulate in North America and elsewhere.[1], [2], [3], [4], [5] Initial doses of a vaccine to prevent pH1N1 infection first became available starting in early October 2009. At that time, target groups for vaccination were identified by the Centers for Disease Control and Prevention's Advisory Committee for Immunization Practices (ACIP).[6] Targeted age groups differ considerably than those for seasonal influenza vaccine for people 65 years and older. Supply of the pH1N1 vaccine was anticipated to be limited initially, raising questions of prioritization. Consideration of the economic consequences of a vaccination program for pH1N1 can aid decision makers in vaccine allocation decisions by providing information on the relative cost-effectiveness of vaccinating specific age and risk groups.

Most studies using dynamic models suggest that vaccinating school-aged children preferentially over other age groups is the optimal strategy for reducing the health consequences of a future pandemic [7], [8], [9], although one study supports the ACIP prioritization strategy of vaccinating high-risk individuals first.[10] The approach of preferentially vaccinating schoolchildren, however, assumes sufficient vaccine is available for all schoolchildren and that coverage rates among this target group will be high enough to reach coverage levels that would achieve herd effects. Such an approach also makes the assumption that society is willing to accept health risks of vaccine adverse events for school-aged children in return for health benefits to adults and younger children. Given the likelihood that vaccine coverage levels may not be sufficient to achieve herd effects and acknowledging that parent preferences may not favor vaccinating school-aged children as a strategy for protecting other individuals but may favor vaccination of children to prevent illness in their own children, the current study evaluates the cost-effectiveness of pH1N1 vaccination by measuring the health benefits that accrue to the vaccinated individual and does not consider indirect effects of vaccination.

## Methods

**Figure 1:**
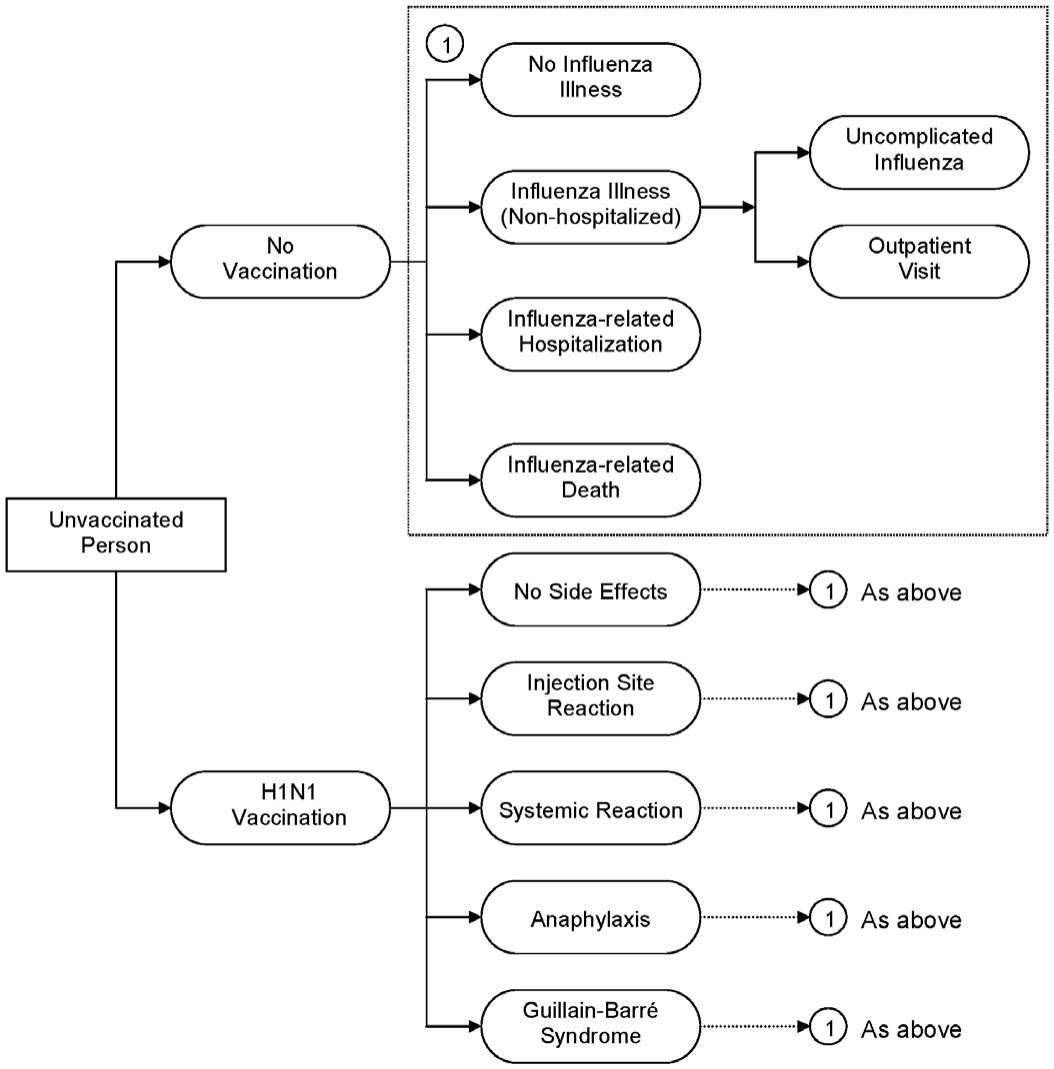
Simplified representation of simulation model. Systemic reaction  =  fever and flu-like symptoms for 24 hours following vaccination.

We used a decision analytic model, built using standard software (TreeAge Pro 2009 Software, release 1.0, Treeage Software, Williamstown, MA), to estimate costs and health outcomes for pH1N1 influenza vaccination compared to no vaccination. A simplified schematic of the decision model is shown in Figure 1. Input parameters were derived from emerging data available for pH1N1 influenza illness in the US in spring/summer 2009, published data, and expert opinion and are described in more detail below (Tables 1, 2) and in supplemental materials (Tables S1, S2). We used a time frame of one year because most costs and consequences related to influenza occur during a single influenza season. However, two key outcomes with longer-term effects, influenza-related deaths and long-term sequelae of influenza-related illness, were included. The analysis used a societal perspective.

**Table 1:** Probabilities of H1N1 influenza illnesses, hospitalizations, and deaths by age and risk status.

Age Group	Overall Attack Rate					Source
	5%	7.6%	15%	21%	30%	
	pH1N1 Influenza Illness Rate					[11]
6–23 months	0.0924	0.1405	0.2772	0.3929	0.5544	
2 y	0.0924	0.1405	0.2772	0.3929	0.5544	
3–4 y	0.0924	0.1405	0.2772	0.3929	0.5544	
5–11 y	0.1085	0.1649	0.3254	0.4613	0.6509	
12–17 y	0.1085	0.1649	0.3254	0.4613	0.6509	
18–49 y	0.0460	0.0699	0.1380	0.1956	0.2760	
50–64 y	0.0158	0.0240	0.0474	0.0671	0.0947	
≥65 y	0.0053	0.0080	0.0159	0.0225	0.0317	
	Incidence of hospitalizations for pneumonia or other respiratory conditions due to pH1N1 influenza per 100,000					[11], [12], [13] 1
6–23 months, LR	95.22	141.45	261.41	424.58	459.98	
2 y, LR	102.08	151.64	280.25	455.22	493.18	
3–4 y, LR	84.63	125.71	232.31	377.28	408.73	
5–11 y, LR	33.70	49.84	91.08	151.30	156.92	
12–17 y, LR	33.70	49.84	91.08	151.30	156.92	
18–49 y, LR	25.60	38.45	73.42	111.43	137.42	
50–64 y, LR	13.25	20.04	39.13	56.73	76.39	
6–23 months, HR	285.93	424.94	786.28	1279.19	1386.34	
2 y, HR	306.55	455.60	843.13	1371.93	1486.91	
3–4 y, HR	338.94	503.79	932.48	1517.75	1645.05	
5–11 y, HR	202.40	299.39	547.71	911.27	945.26	
12–17 y, HR	202.40	299.39	547.71	911.27	945.26	
18–49 y, HR	128.07	192.39	367.65	558.41	689.01	
50–64 y, HR	66.26	100.26	195.82	283.95	382.55	
≥65 y, all	14.08	21.23	42.03	59.96	83.16	
	Incidence of pH1N1 influenza death per 100,000					[11], [12], [13]
6–23 months, LR	0.0843	0.1252	0.2313	0.3754	0.4066	
2 y, LR	0.0904	0.1342	0.2480	0.4024	0.4359	
3–4 y, LR	0.0749	0.1113	0.2056	0.3336	0.3614	
5–11 y, LR	0.0299	0.0441	0.0807	0.1340	0.1389	
12–17 y, LR	0.0299	0.0441	0.0807	0.1340	0.1389	
18–49 y, LR	2.8483	4.2772	8.1666	12.3926	15.2811	
50–64 y, LR	1.6245	2.4575	4.7975	6.9538	9.3638	
6–23 months, HR	0.2530	0.3757	0.6939	1.1262	1.2198	
2 y, HR	0.2712	0.4027	0.7439	1.2072	1.3077	
3–4 y, HR	0.2998	0.4452	0.8223	1.3346	1.4456	
5–11 y, HR	0.1791	0.2649	0.4839	0.8037	0.8336	
12–17 y, HR	0.1791	0.2649	0.4839	0.8037	0.8336	
18–49 y, HR	14.2422	21.3880	40.8398	61.9785	76.4289	
50–64 y, HR	8.1227	12.2882	23.9900	34.7736	46.8276	
≥65 y, all	1.8141	2.7346	5.4138	7.7222	10.7092	

LR  =  lower-risk; HR  =  higher-risk.

1 Data from seasonal influenza illness was used to estimate the ratio of high risk to low risk based on expert opinion that although the incidence for pH1N1 and seasonal influenza varied substantially by age, the conditional probability of influenza-related complications for high risk and low risk patients would likely be similar for pH1N1 and seasonal (i.e., within an age group, high risk patients would be more likely to experience influenza-related complications than low-risk patients for both pH1N1 and seasonal influenza).

**Table 2:** Effectiveness and costs associated with pH1N1 vaccination by age and risk status (inactivated vaccine).

	Vaccine Effectiveness			
Age Group	Most Likely	Minimum	Maximum	Source
6 mo–17 y	0.690	0.40	0.90	[19]
18–64 y	0.690	0.30	0.90	[18], [19]
≥65 y	0.600	0.30	0.90	[20], [21]

	Vaccination Costs^2^			
	Mass Vaccination Setting	Mid-Range^3^	Physician Office Setting^4^	[13], [33], [58], [59]
6–23 months	$68.67	-	$93.32	
2 y	$68.67	-	$93.32	
3–4 y	$68.67	-	$93.32	
5–11 y	$39.80	$53.18	$93.32	
12–17 y	$19.90	$25.72	$43.18	
18–49 y, LR	$34.33	$35.30	$37.89	
50–64. LR	$34.33	$35.07	$37.06	
18–49 y, HR	$34.33	$36.11	$37.89	
50–64. HR	$34.33	$35.97	$37.06	
≥65 y, all	$34.33	$33.05	$31.77	

LR  =  lower-risk; HR  =  higher-risk.^1^

1 Individuals were defined to be at higher risk for influenza-related complications due to underlying medical conditions, which include chronic pulmonary and significant cardiac conditions and other recognized high-risk conditions.[6]

2 Total vaccination costs include the cost for the vaccine (1 dose for persons aged 10 years or older and 2 doses for children aged 6 months to 9 years), administration costs, and time costs as appropriate. Cost of the vaccine dose was based on the contracts negotiated by the Biomedical Advanced Research and Development Authority for pH1N1 vaccine in 2009 (average cost: $8.60 per dose). Administration costs are assumed to be $11.30 per dose in a mass vaccination and either $13.71 for administration during an existing visit to a clinician in a physician office setting or $20.92 for administration during an extra physician office setting visit based on Medicare payment rates.[33] For vaccination cost estimates in the mass vaccination setting, (travel and vaccination) time costs included 12 minutes for waiting and vaccination time[13] at the clinic and 30 minutes of travel time for all adult age groups and for age groups less than 5 years to account for parent time costs. Children 5–17 years in the mass vaccination setting are assumed to be vaccinated in a school setting and therefore no parent time costs were included.

3 Assumes a mix of mass vaccination and physician office. For the mixed setting, the proportion of persons vaccinated in a mass vaccination setting vs. a physician's office was varied by age. Time costs are always included for parents of children younger than 5 years of age assuming that a parent will need to be present for vaccination of young children in any setting. See supporting information for additional details.

4 For the physician office setting, the proportion of persons needing one vs. two extra physician visits to accommodate vaccination was varied by age. Time costs were included for parents of children <18 and for adults for each extra visit required for vaccination. For the physician office setting, we include 60 minutes of time for travel, waiting, and vaccination time for either the vaccinee or the parent, which assumes a streamlined setting is used for vaccination.

### Target population

The model includes cohorts of children and adults aged 6 months and older stratified by age and risk of complications. Age groups were: 6–23 month, 24–35 month (2 yrs), 3–4 years, 5–11 years, 12–17 years, 18–49 years, 50–64 years, and 65 years and older. Each age group was then divided into two risk-based groups, higher risk and lower risk, except for age 65 years and older who are all assumed to be at higher risk for complications. Higher risk groups were defined using conditions identified by CDC as placing individuals at higher risk for medical complications of influenza illness.[6]

### Natural history of influenza

Probabilities of hospitalization and death following pH1N1 illness were derived from emerging data for pH1N1 in spring/summer 2009.[11](Table 1) Input values for probabilities of other influenza-related outcomes were based on previously established estimates for seasonal influenza, such as probability of seeking medical attention during an episode of influenza illness, other complications treated on an outpatient setting, and long-term sequelae following hospitalization.[12], [13] The range of possible values for probability of pH1N1 influenza illness during a single season was varied from 5% to 30% to reflect the limited data on possible overall pH1N1 illness attack rates with intermediate probabilities of 7.6%, 15%, and 21%. One intermediate probability, 21%, represents the most recent estimate for the pH1N1 pandemic derived from Shrestha et al (2011).[14] The probability of 7.6% represents an average non-pandemic influenza season.

### Costs of influenza-related health events

Costs included direct medical costs for influenza events, including physician visits, over-the-counter and prescription medications, diagnostic tests, hospitalizations, and long-term sequelae, based on established costs for seasonal influenza.[12], [13], [15], [16] Direct medical costs were adjusted to 2009 dollars using the medical component of the Consumer Price Index.[17]

### Vaccination assumptions

Pandemic H1N1 vaccine effectiveness was assumed to have similar effectiveness as for seasonal influenza vaccine based on preliminary studies of 2009 pH1N1 vaccine immunogenicity which shows immune responses comparable to seasonal influenza vaccine.[18], [19], [20], [21] Base case (range) vaccine effectiveness was assumed to be 69% (40%–90%) for individuals aged 6 months to 17 years, 69% (30%–90%) for those 18 to 64 years and 60% (30%–90%) for those 65 years and older. (Table 2) Vaccination-related adverse events were assumed to be consistent with rates for seasonal influenza vaccine. Injection site reactions, systemic reactions, anaphylaxis, and Guillain Barré syndrome varied by age and are listed in Table S1. Incidence of Guillain Barré syndrome was based on data from seasonal influenza vaccine[22] and varied in sensitivity analyses to reflect rates observed in the 1976 swine flu vaccination program.[23], [24], [25], [26], [27], [28], [29], [30], [31], [32].

Vaccination-related costs included cost of pH1N1 vaccine doses, administration fees, and time costs (for parents or patients, depending on the age of the vaccinee).(Tables 2, S1, S2) We assumed full vaccination required 2 doses for individuals aged 6 months to 9 years and 1 dose for individuals aged 10 years and older. Administration costs varied by setting: lower for mass vaccination clinic and higher for the physician office setting.[33] For the physician office setting, the proportion of persons vaccinated at an existing or vaccination-specific visit varied by age. Assumptions for mid-range costs use data on vaccination setting by age from seasonal influenza vaccination for adults and assume a mix of vaccination settings.[34] The proportion of individuals receiving vaccination in a mass vaccination or physician office setting varied by age and risk group. Assumptions for mid-range costs for children also vary by age. 75% of school-aged children were assumed to be vaccinated in a school-located setting. Children younger than 5 years were assumed to be vaccinated in the physician office setting. Costs of adverse events were based on costs of adverse events associated with inactivated influenza vaccine.[15] Time costs for vaccination time for adults and parents of vaccinated children were also included.

### Health outcomes and quality adjustments

The primary health outcome for the analysis is the quality-adjusted life year (QALY). A QALY attempts to measure a patient's physical health and well being including, among other factors, the ability to engage in “normal,” everyday activities. QALYs lost to a disease or condition, therefore, measure the overall reduction in a patient's well being, or health-related quality of life, due to an episode of disease and its consequences (which may last a life time). We obtained the loss in QALYs associated with each influenza-related health event from published studies and primary data.[35], [36], [37] In these studies, respondents were asked how much of their own lifetime they would be willing to trade in order to avoid a case of influenza-related illness or a vaccination-related adverse event (i.e., a time-tradeoff valuation). (Tables S1, S2)

### Cost-effectiveness Analysis

The main endpoint for the study was the incremental cost-effectiveness ratio (ICER) calculated by dividing the net costs by the net health benefits (as measured via QALYs) for vaccination compared to no vaccination. An intervention is defined as cost-saving if the intervention decreases dollar costs and also results in an increase in QALYs. An intervention is defined as cost-effective if it results in an increase in both costs and QALYs and the resulting ratio is less than a determined threshold.[38] The primary analysis explored a range of values for pH1N1 illness rates and vaccination costs due to uncertainty regarding the severity of the pH1N1 influenza season and preferred vaccination settings. All costs and health effects lasting more than 1 year were discounted at 3% per year.

Considerable uncertainty exists regarding the most likely setting for pH1N1 vaccination (i.e., mass vaccination clinics compared with physician offices). Therefore the primary analysis includes a mass vaccination setting, a physician office setting, and a “mid-range” cost setting that assumes a proportion of individuals is vaccinated in each setting. This proportion varies by age. The primary analysis assumes that some individuals will receive pH1N1 vaccination at an existing physician visit which would be associated with lower administration costs. An existing visit is defined as a visit previously-scheduled for a purpose other than pH1N1 vaccination. In the sensitivity analysis, we include a more conservative scenario in which all individuals are vaccinated in the physician office setting and require a vaccine-specific visit.

Sensitivity analyses explored changes in key variables including the number of doses required for vaccination, costs of vaccination, vaccination-related adverse events, and influenza-related hospitalization rates. A sensitivity analysis explored the change in cost-effectiveness ratios if only one dose were required for children younger than 10 years. Some studies have suggested higher costs of influenza vaccination in the physician office setting[39], [40], therefore, we also conducted a sensitivity analysis that assumed higher administration costs. Due to concerns that swine influenza vaccination in 1976–1977 may have been associated with Guillain-Barré syndrome, sensitivity analyses included varying the probability of Guillain-Barré syndrome following vaccination over a wide range of possible values, from rates that may occur with seasonal influenza vaccine (1 per million) to rates observed with 1976 swine influenza vaccine (1 per 100,000).

The primary analysis assumed timely pH1N1 vaccination prior to the start of the outbreak. A scenario analysis evaluated initiation of vaccination after the start of a hypothetical influenza season. Each week after the start of the season that vaccination was initiated was assumed to reduce the protective effect of the vaccine according to the expected distribution of cases over a hypothetical 16-week influenza season assuming peak at 9 weeks and 70% of cases occur between weeks 7–10.(Table S3) [41]

## Results

Assuming a 21% overall attack rate and assuming that persons were fully vaccinated prior to the start of the outbreak, pH1N1 vaccination was cost-saving for all high-risk subgroups ages 6 months to 64 years. For low-risk subgroups 6 months to 64 years, pH1N1 vaccination required a net investment for a return in health benefits. The cost-effectiveness ratios for these subgroups ranged from $5,000–$18,000/QALY depending on age and risk. Cost-effectiveness ratios were least favorable for persons aged 65 years and older.(Table 3)

**Table 3:** Incremental cost-effectiveness ratios, $/QALY (2009 US dollars).^1^

	Vaccination Costs		
Age Group	Mass Vaccination Setting	Mid-Range^2^	Physician Office Setting^3^
**a. 5% influenza illness attack rate**			
6–23 months, LR	$37,896	-	$54,182
2 y, LR	$42,190	-	$60,278
3–4 y, LR	$55,321	-	$78,481
5–11 y, LR	$41,664	$57,471	$106,152
12–17 y, LR	$26,049	$35,998	$65,804
18–49 y, LR	$38,393	$40,825	$43,257
50–64 y, LR	$113,660	$119,678	$123,690
≥65 y, all	$255,920	$245,729	$235,537
6–23 months, HR	$3,515	$10,104	$10,104
2 y, HR	$3,709	$10,668	$10,668
3–4 y, HR	$440	$8,218	$8,218
5–11 y, HR	Cost-saving	$3,130	$20,852
12–17 y, HR	Cost-saving	Cost-saving	$4,668
18–49 y, HR	Cost-saving	Cost-saving	Cost-saving
50–64 y, HR	$1,795	$3,357	$4,398
**b. 7.6% influenza illness attack rate (comparable to average attack rate for seasonal influenza)**			
6–23 months, LR	$22,374	-	$33,339
2 y, LR	$25,312	-	$37,492
3–4 y, LR	$33,892	-	$49,486
5–11 y, LR	$25,515	$36,419	$69,130
12–17 y, LR	$14,859	$21,581	$41,475
18–49 y, LR	$22,552	$24,163	$25,775
50–64 y, LR	$69,691	$73,615	$76,230
≥65 y, all	$158,736	$152,171	$145,607
6–23 months, HR	Cost-saving	$1,589	$1,589
2 y, HR	Cost-saving	$1,820	$1,820
3–4 y, HR	Cost-saving	Cost-saving	Cost-saving
5–11 y, HR	Cost-saving	Cost-saving	$7,351
12–17 y, HR	Cost-saving	Cost-saving	Cost-saving
18–49 y, HR	Cost-saving	Cost-saving	Cost-saving
50–64 y, HR	Cost-saving	Cost-saving	Cost-saving
**c. 15% influenza illness attack rate**			
6–23 months, LR	$7,702	$13,638	$13,638
2 y, LR	$9,360	$15,954	$15,954
3–4 y, LR	$13,637	$22,079	$22,079
5–11 y, LR	$10,240	$16,207	$34,109
12–17 y, LR	$4,275	$7,953	$18,989
18–49 y, LR	$7,662	$8,502	$9,342
50–64 y, LR	$28,976	$30,960	$32,282
≥65 y, all	$69,236	$66,011	$62,787
6–23 months, HR	Cost-saving	Cost-saving	Cost-saving
2 y, HR	Cost-saving	Cost-saving	Cost-saving
3–4 y, HR	Cost-saving	Cost-saving	Cost-saving
5–11 y, HR	Cost-saving	Cost-saving	Cost-saving
12–17 y, HR	Cost-saving	Cost-saving	Cost-saving
18–49 y, HR	Cost-saving	Cost-saving	Cost-saving
50–64 y, HR	Cost-saving	Cost-saving	Cost-saving
**d. 21% influenza illness attack rate**			
6–23 months, LR	$1,053	$4,711	$4,711
2 y, LR	$2,131	$6,194	$6,194
3–4 y, LR	$4,459	$9,661	$9,661
5–11 y, LR	$2,893	$6,486	$17,265
12–17 y, LR	CS	$1,399	$8,043
18–49 y, LR	$2,115	$2,667	$3,220
50–64 y, LR	$15,942	$17,305	$18,214
≥65 y, all	$42,901	$40,659	$38,417
6–23 months, HR	Cost-saving	Cost-saving	Cost-saving
2 y, HR	Cost-saving	Cost-saving	Cost-saving
3–4 y, HR	Cost-saving	Cost-saving	Cost-saving
5–11 y, HR	Cost-saving	Cost-saving	Cost-saving
12–17 y, HR	Cost-saving	Cost-saving	Cost-saving
18–49 y, HR	Cost-saving	Cost-saving	Cost-saving
50–64 y, HR	Cost-saving	Cost-saving	Cost-saving
**e.** **30% influenza illness attack rate**			
6–23 months, LR	$234	$3,611	$3,611
2 y, LR	$1,240	$4,991	$4,991
3–4 y, LR	$3,328	$8,130	$8,130
5–11 y, LR	$2,495	$5,960	$16,352
12–17 y, LR	Cost-saving	$1,044	$7,450
18–49 y, LR	$93	$541	$989
50–64 y, LR	$8,528	$9,537	$10,211
≥65 y, all	$25,910	$24,302	$22,694
6–23 months, HR	Cost-saving	Cost-saving	Cost-saving
2 y, HR	Cost-saving	Cost-saving	Cost-saving
3–4 y, HR	Cost-saving	Cost-saving	Cost-saving
5–11 y, HR	Cost-saving	Cost-saving	Cost-saving
12–17 y, HR	Cost-saving	Cost-saving	Cost-saving
18–49 y, HR	Cost-saving	Cost-saving	Cost-saving
50–64 y, HR	Cost-saving	Cost-saving	Cost-saving

LR  =  lower-risk; HR  =  higher-risk.

1 Vaccination in each setting is compared to no vaccination.

2 Assumes a mix of mass vaccination and physician office for individuals aged 5 years and older. For the mixed setting, the proportion of persons vaccinated in a mass vaccination setting vs. a physician's office was varied by age. For children younger than 5 years of age, the assumption is that very few children will be vaccinated in the mass vaccination setting, therefore the physician office setting is considered to be the primary setting.

3 For the physician office setting, the proportion of persons needing one vs. two extra physician visits to accommodate vaccination was varied by age. Time costs were included for parents of children <18 and for adults for each extra visit required for vaccination.

Assuming a higher overall attack rate of 30%, cost-effectiveness ratios were less than $30,000/QALY for all age and risk groups for the full range of vaccination costs. Vaccination remained cost-saving for all high-risk subgroups. For lower attack rates, cost-effectiveness results were less favorable. Assuming an attack rate similar to that for an average influenza season (7.6%), vaccination was no longer cost-saving for all high-risk subgroups.(Table 3)

Results were sensitive to changes in the number of doses required for children, costs of vaccination, and timing of vaccine delivery. Requiring only one vaccine dose for children resulted in more favorable cost-effectiveness ratios compared to two doses.(Table S4) Cost-effectiveness ratios were 72-89% lower for lower-risk children and vaccination remained cost-saving for high-risk children.(Table S4)

Higher vaccination costs were associated with less favorable cost-effectiveness ratios for vaccination. If we assume that all adults will receive vaccination in the physician office setting at a vaccine-specific visit (our most conservative setting for vaccination costs), cost-effectiveness ratios become less favorable by 26–44% for lower-risk individuals, but remained cost-saving for higher-risk adults who were younger than 65 years of age. Assuming the cost per dose to be twice that in the primary analysis resulted in cost-effectiveness ratios up to 68% higher than the primary analysis, but cost-effectiveness ratios remained below $100,000/QALY. Higher administration costs in the physician office setting would result in higher cost-effectiveness ratios (up to 18% higher); vaccination remained cost-saving for high-risk subgroups.(Table S4)

Results were not sensitive to changes in the probability of Guillain-Barré syndrome following vaccination when varying the probability up to 1 in 100,000. Results were also not sensitive to an increase in hospitalization rates for high-risk individuals based on emerging pH1N1 data; vaccination remained cost-saving for high risk groups.

Timing of vaccination affects the cost-effectiveness of vaccination and depended on when vaccination occurred and if the age group under consideration required one or two doses. For subgroups requiring two doses and vaccination is not initiated until the third week of the season, vaccination remains cost-saving for high-risk children and cost-effectiveness ratios remain below $10,000/QALY for lower-risk children assuming a hypothetical 16-week flu season and normalized epidemic curve of illness. If vaccination is initiated beyond the tenth week into a hypothetical 16-week influenza season, the cost-effectiveness ratios become less favorable for subgroups requiring two doses. If vaccination is initiated beyond the fifteenth week, no vaccination becomes the preferred strategy from an economic perspective. For age groups requiring one dose, cost-effectiveness ratios increase markedly if vaccination is initiated at the ninth or tenth week of the epidemic. Results are similar for adults with the exception of individuals 65 years and older, for whom cost-effectiveness ratios are overall less favorable.(Table 4)

**Table 4:** Scenario analysis for delayed vaccine availability (by week)^1^, assuming 16-week influenza epidemic.

a. Age groups that require two doses (assumes 5 weeks to full protection; e.g., “Week 0” assumes that children are fully vaccinated prior to the first week of the outbreak)								
Week of Full Immuniz-ation^2^	6–23 m, HR	2 y, HR	3–4 y, HR	5–11 y, HR	6–23 m, LR	2 y, LR	3–4 y, LR	5–11 y, LR
0	CS	CS	CS	CS	$4,711	$6,194	$9,661	$6,486
1	CS	CS	CS	CS	$4,712	$6,195	$9,662	$6,487
2	CS	CS	CS	CS	$4,716	$6,199	$9,667	$6,490
3	CS	CS	CS	CS	$4,726	$6,210	$9,682	$6,501
4	CS	CS	CS	CS	$4,761	$6,248	$9,730	$6,537
5	CS	CS	CS	CS	$4,876	$6,374	$9,891	$6,656
6	CS	CS	CS	CS	$5,256	$6,790	$10,419	$7,045
7	CS	CS	CS	CS	$6,506	$8,156	$12,158	$8,329
8	CS	CS	CS	CS	$10,624	$12,658	$17,887	$12,556
9	CS	CS	CS	CS	$24,190	$27,489	$36,757	$26,482
10	$16,122	$16,921	$15,151	$8,635	$68,912	$76,383	$98,973	$72,398
11	$76,497	$79,656	$84,715	$64,147	$216,721	$237,985	$304,628	$224,209
12	$277,592	$288,617	$316,434	$249,119	$709,347	$776,645	$990,353	$730,798
13	$965,431	$1,003,421	$1,109,230	$882,681	$2,400,000	$2,638,889	$3,357,143	$2,474,642
14	$3,551,412	$3,691,592	$4,092,708	$3,276,700	$8,727,273	$10,555,556	$13,428,571	$9,200,000
15	$18,204,581	$18,948,070	$21,085,287	$17,213,376	$48,500,000	$95,000,000	$94,000,000	$46,000,000
16	NV	NV	NV	NV	NV	NV	NV	NV
CS = Cost Saving; NV = No Vaccination is the preferred alternative as health risks of vaccination outweigh health benefits for these conditions.								

b. Age groups that require one dose only (assumes that vaccination occurred 2 weeks prior to the “Week of Full Immunization”^3^)							
Week of Full Immuniz-ation^4^	12–17 y, HR	18–49 y, HR	50–64 y, HR	12–17 y, LR	18–49 y, LR	50–64 y, LR	≥65 y, all
0	CS	CS	CS	$1,399	$2,667	$17,305	$40,659
1	CS	CS	CS	$1,400	$2,668	$17,037	$40,663
2	CS	CS	CS	$1,402	$2,670	$17,314	$40,676
3	CS	CS	CS	$1,409	$2,679	$17,337	$40,720
4	CS	CS	CS	$1,433	$2,706	$17,411	$40,864
5	CS	CS	CS	$1,513	$2,797	$17,654	$41,338
6	CS	CS	CS	$1,776	$3,095	$18,456	$42,902
7	CS	CS	CS	$2,641	$4,078	$21,099	$48,063
8	CS	CS	CS	$5,491	$7,318	$29,836	$65,185
9	CS	CS	CS	$14,881	$18,037	$58,951	$122,985
10	CS	CS	$13,120	$45,841	$53,880	$158,715	$330,107
11	$33,027	$21,073	$99,555	$148,216	$178,107	$536,073	$1,266,867
12	$156,273	$134,121	$423,416	$489,987	$668,719	$2,752,458	$71,159,030
13	$578,619	$586,517	$2,232,044	$1,668,217	$4,418,475	NV	NV
14	$2,177,431	4,392,915	NV	$6,500,000	NV	NV	NV
15	$11,577,092	NV	NV	$27,000,000	NV	NV	NV
16	NV	NV	NV	NV	NV	NV	NV
CS = Cost Saving; NV = No Vaccination is the preferred alternative as health risks of vaccination outweigh health benefits for these conditions.							

LR  =  lower-risk; HR  =  higher-risk.

1 21% influenza illness attack rate, mid-range costs assumption.

2 For children <10 y needing two doses, vaccination would need to have been initiated ≥5 weeks earlier assuming 3 weeks between doses and 2 weeks after the second dose for peak antibody response. http://www.cdc.gov/h1n1flu/vaccination/public/vaccination_qa_pub.htm For children ≥10 y, only one dose is needed.; vaccination would need to have been initiated ≥2 weeks earlier for peak antibody response. Intermediate protection in between weeks 3 and 5 for children who have received the first of two doses is conservatively assumed to be zero.

3 Vaccination is assumed to require 2 weeks to achieve full protection.

4 For individuals ≥10 y, only one dose is needed. Vaccination would need to have been initiated ≥2 weeks earlier for peak antibody response.

## Discussion

The Advisory Committee on Immunization Practices (ACIP) recommended initial target groups for vaccination against pH1N1 influenza, which include pregnant women, household contacts of infants younger than 6 months, health care and emergency medical personnel, persons aged 6 months through 24 years, and persons aged 25 through 64 years at higher risk for influenza-related complications.[6] Using the assumptions from the primary analysis, we find that the cost-effectiveness of vaccinating children and high-risk working-age adults against pH1N1 is within the range of cost-effectiveness for other vaccines recently recommended by ACIP, including seasonal influenza vaccine[12], pneumococcal conjugate vaccine[36], and HPV[42], [43], [44], [45]. We also find that pH1N1 vaccination is cost-saving for high-risk individuals less than 65 years under a wide range of assumptions.

We did not include separate analyses for some of the initial target groups for recommendation, such as health care workers, pregnant women, or household contacts of infants younger than 6 months. However, health care workers and pregnant women would be included as part of the overall high-risk and low-risk group calculations. Given increased exposure of health care workers influenza and increased risk of complications of pH1N1 in pregnant women[46], cost-effectiveness ratios would likely be at least as favorable as for corresponding target groups as defined by age and risk category.

The live attenuated formulation is not explicitly included in the current analysis. Live attenuated vaccine for seasonal influenza may be more effective than inactivated vaccine for young children[47], yet recent data suggest that inactivated vaccines may be more effective for young adults[48]. However, there are no data on the effectiveness of live attenuated pH1N1 vaccine by age group and it is possible that this may differ from that for seasonal vaccine.

Emerging data on the epidemiology of pH1N1 influenza virus infection were used in the simulation model where available, but some assumptions were based on data from seasonal influenza. These include the probability that an individual will seek medical attention if they experience influenza-like illness. If individuals are more likely to seek medical attention if they think they have pH1N1 infection, these results represent a conservative approach to assessing the cost-effectiveness of vaccination. Similarly, if the costs of treating pH1N1 infection are substantially higher than for seasonal influenza, the results of this analysis will also be conservative. We did not consider any costs related to potential school closures or mandated absences from school or work due to illness. If these costs were appreciable, the results would be more favorable for vaccination if these additional costs of illness were included.

### Policy Implications

This analysis differs from dynamic models, which model the indirect effects of vaccination. One such model suggested that vaccinating school-aged children and adults between the ages of 30 and 40 would be most cost-effective.[7] Dynamic models simulate the transmission of infection among individuals and estimate the reduction in infections as a result of reduced transmission among unvaccinated age groups (e.g., vaccinating school children will reduce transmission to individuals of other ages and result in indirect effects of reducing illness and deaths in infants and the elderly). The current analysis intentionally excludes possible indirect effects of vaccination and restricts the primary analysis to the costs and health benefits to the vaccinated individual. Required coverage rates to generate herd effects for pH1N1 are unknown and substantial uncertainty exists for the required minimum coverage level for seasonal influenza[49], [50], [51]. Even if higher coverage rates are attained or pH1N1 vaccination compared to seasonal influenza vaccination, the effect of herd immunity is uncertain.[49], [50], [51] Another rationale for restricting the analysis to individual-level benefits relates to societal preferences. A policy of vaccinating school children to prevent illness in other age groups assumes that public preferences are consistent with trading off the health and well-being of school-aged children (in the form of risk for vaccination-related adverse events) to protect people in other age groups. Evidence suggesting that societal preferences may be more consistent with prioritizing child health over adult health could clearly support the vaccination of children if the expected benefits to the child outweighed the potential costs and risks but the decision to vaccinate a child may only consider benefits and risks to the vaccinated child.[37] Given that the inclusion of herd effects would result in an increase in the health benefits associated with vaccination, an extension of the current analysis to include herd effects would result in more favorable cost-effectiveness results.

The costs of vaccination have a substantial impact on cost-effectiveness results. Uncertainty exists as to the proportion of individuals likely to get vaccinated in each type of setting. Since costs of vaccination will vary with setting, these are key assumptions for the analysis. For higher attack rate scenarios, cost-effectiveness ratios vary about 10% across settings, however, if most people are vaccinated in the physician office setting and require a vaccine-specific visit, vaccination costs will be higher and associated cost-effectiveness ratios will be less favorable. On the other hand, if only one dose is required for vaccinating children, the associated costs will be lower and cost-effectiveness ratios will be more favorable. Recent data from the 2009–2010 pH1N1 vaccination season indicate that a mix of settings was used for vaccination against pH1N1 influenza.[52] Results of sensitivity analysis varying the costs of vaccination could provide useful information for future decision making given the sensitivity of the results to this input parameter.

Initiation of vaccination after the start of the season will affect the cost-effectiveness of vaccination depending on the timing of availability relative to the start, duration, and intensity of influenza activity in a community. For children who require two doses, vaccination may not be cost-effective if vaccine is delivered such that full protection is not achieved until after the 8^th^ week (the peak) of a hypothetical influenza season. For adults and children requiring only one dose, results are similar but the timing of vaccination required will differ since only one dose is assumed to be required for full protection. Cost-effectiveness results would differ if additional pandemic waves caused by a similar virus were to occur within the same vaccination year, or if vaccination later during a single pandemic wave provided some beneficial immunologic priming for subsequent vaccination against a drifted influenza virus, or if the pattern of disease during the pandemic wave does not conform to our model of a hypothetical season.

### Comparison to Other Economic Studies of pH1N1 Vaccination

Estimates of the economic impact of vaccination are available for the US and other countries. In the US, Beigi et al. evaluate the economic value of vaccinating pregnant women, a very high risk group not included in our analysis, and report favorable cost-effectiveness ratios for maternal influenza vaccination.[53] Khazeni et al. (2009) evaluate the cost-effectiveness of vaccination against pandemic influenza A (H1N1) for a major US metropolitan city and early vaccination to be cost-saving.[54] Lee et al. (2010) estimate averted lost productivity costs for an employee population but do not report results using an economic metric such as an incremental cost-effectiveness ratio making it difficult to compare these results with those from our study.[55] Sander et al (2010) find the cost-effectiveness of a mass immunization program for pandemic H1N1 to be favorable but this study does not evaluate the cost-effectiveness of individual age and risk groups.[56] Baguelin et al. (2010) use a dynamic model to evaluate vaccination against pandemic influenza A(H1N1) in England.[57] Our analysis complements other published analyses both for the US and abroad by evaluating the cost-effectiveness of individual age and risk groups and considering explicitly the effects of delay in vaccination on cost-effectiveness. While it is difficult to directly compare cost-effectiveness analyses across countries due to differences in costs of services and level of intensity of care, results from other countries were consistent with ours in estimating favorable cost-effectiveness for mass vaccination in England and Canada. Our analysis provides additional information to the previously published studies by providing incremental cost-effectiveness ratios for separate age and risk groups relevant to the US setting. In addition, we explored sensitivity analyses relevant to the US decision maker perspective for costs of vaccination, influenza illness rates, and delays in vaccination using the best available information at the time of the pandemic.

None of the published studies reviewed above appear to have accounted for costs or health consequences potentially associated with H1N1 vaccination, except for the Khazeni et al and Beigi et al. studies. The exclusion of vaccination-related adverse events would yield more favorable results for vaccination compared with a more comprehensive analysis that included adverse events. Both costs and health effects of potential vaccine adverse events are explicitly accounted for in our analysis. Additionally, outside the US, adjuvanted vaccine was typically used and this formulation could result in a different risk profile than non-adjuvanted vaccine. The study by Khazeni et al. assumed the use of adjuvanted vaccine for a US setting and is not directly comparable to our study due to assumed differences in effectiveness and side effect profile between the two formulations.

### Conclusions

Vaccination for pH1N1 influenza for children and young adults is cost-effective compared to other preventive health interventions under a wide range of scenarios. Delayed availability of pH1N1 vaccine results in less favorable cost-effectiveness results. A vaccination program for pH1N1 influenza for target groups can be justified from an economic perspective when indirect benefits are not considered and assuming that vaccine supplies are sufficient. Additional economic and health benefits beyond direct benefits would only add to the cost-effectiveness of pandemic influenza vaccination.

## Supporting Information

Table S1 **Additional model input parameters, 6 months –17 years.**
(DOCX)

Table S2 **Additional model input parameters, 18 years and older.**
(DOCX)

Table S3 **Cumulative cases by week for hypothetical 16-week epidemic.**
(DOCX)

Table S4 **Sensitivity analyses with varying vaccination cost assumptions, $/QALY.**
(DOCX)
