# Supplementary material for: Cost-Effectiveness of 2009 Pandemic Influenza A(H1N1) Vaccination in the United States
Source: PLoS One. 2011 Jul 29;6(7):e22308. doi: 10.1371/journal.pone.0022308 (PMC3146485; doi:10.1371/journal.pone.0022308)
Supplement: Table S1 — Additional model input parameters, 6 months –17 years. (DOCX) [file pone.0022308.s001.docx]

Table S1. Additional model input parameters, 6 months – 17 years.

| Variable | Most Likely Estimate | Range for Sensitivity Analysis | Source |
| --- | --- | --- | --- |
| Probability of an outpatient visit for child with influenza illness^1,2^ |  |  | [[1](#_ENREF_1),[2](#_ENREF_2),[3](#_ENREF_3)] |
| 6-23 months | 0.5 | 0.17-0.83 |  |
| 2 years | 0.47 | 0.15-0.81 |  |
| 3-4 years | 0.43 | 0.12-0.78 |  |
| 5-11 years | 0.28 | 0.11-0.5 |  |
| 12-17 years | 0.24 | 0.06-0.5 |  |
| Probability of otitis media for a child with medically attended influenza illness^3^ |  |  | [[4](#_ENREF_4),[5](#_ENREF_5),[6](#_ENREF_6)], expert panel |
| 6-23 months | 0.63 | 0.33-0.8 |  |
| 2 years | 0.58 | 0.27-0.8 |  |
| 3-4 years | 0.39 | 0.17-0.6 |  |
| 5-11 years | 0.23 | 0.05-0.5 |  |
| 12-17 years | 0.15 | 0.01-0.4 |  |
| Probability of non-hospitalized pneumonia or other outpatient complication for child with medically-attended influenza illness^4,5^ |  |  | [[2](#_ENREF_2),[3](#_ENREF_3)], expert panel |
| 6-23 months | 0.2 | 0.04-0.5 |  |
| 2 years | 0.15 | 0.02-0.4 |  |
| 3-4 years | 0.15 | 0.02-0.4 |  |
| 5-11 years | 0.11 | 0.02-0.3 |  |
| 12-17 years | 0.08 | 0.01-0.2 |  |
| Probability of long-term sequelae following influenza-related hospitalization^6,7^ | 0.01 | 0.001-0.03 | Expert panel |
| Probability of medically-attended vaccination-related adverse events |  |  |  |
| Injection site reaction |  |  |  |
| 6-23 months | 0.008 | 0.002-0.017 | [[7](#_ENREF_7)] |
| 2 years | 0.003 |  |  |
| 3-4 years | 0.002 |  |  |
| 5-11 years | 0.001 |  |  |
| 12-17 years | 0.0003 |  |  |

^1^ Estimates for healthy children shown in table. Probabilities are estimated to be twice as high for children at high risk for influenza-related complications.

^2^ Range for sensitivity analysis determined by expert opinion.

^3^ This was the conditional probability of otitis media given that a child had pH1N1 illness.

^4^ Estimates for healthy children shown in table. Probabilities are estimated to be twice as high for children at high risk for influenza-related complications.

^5^ This was the conditional probability of non-hosptiatlized pneumonia given that a child had pH1N1 illness.

^6^ E.g., acute necrotizing encephalopathy with irreversible neurologic damage.

^7^ This was the conditional probability of long-term sequelae given that a child had pH1N1 illness.

| Variable | Most Likely Estimate | Range for Sensitivity Analysis | Source |
| --- | --- | --- | --- |
| Systemic reaction (fever)^8^ |  |  |  |
| 6-23 months | 0.013 | 0.001-0.025 | [[8](#_ENREF_8)] |
| 2 years | 0.011 |  |  |
| 3-4 years | 0.009 |  |  |
| 5-11 years | 0.004 |  |  |
| 12-17 years | 0.003 |  |  |
| Anaphylaxis | 0.000001 | 0-0.000002 | [[9](#_ENREF_9)] |
| Guillain-Barré syndrome (medically attended within 6 weeks of vaccination) | 0.000001 | 0 - 0.000115 | [[10](#_ENREF_10),[11](#_ENREF_11),[12](#_ENREF_12),[13](#_ENREF_13),[14](#_ENREF_14),[15](#_ENREF_15),[16](#_ENREF_16),[17](#_ENREF_17),[18](#_ENREF_18),[19](#_ENREF_19)] |
| Influenza-related costs |  |  |  |
| OTC medications^9^ | $3 |  | [[20](#_ENREF_20),[21](#_ENREF_21),[22](#_ENREF_22)], expert panel |
| Physician visit for uncomplicated influenza^10^ | $42 | $11-110 | [[23](#_ENREF_23)]^11^ |
| Physician visit for otitis media |  |  | [[23](#_ENREF_23)]^12^ |
| 6-23 months | $98 | $29-249 |  |
| 2-4 years | $105 | $29-253 |  |
| 5-17 years | $119 | $39-310 |  |
| Physician visit for non-hospitalized pneumonia |  |  | [[23](#_ENREF_23)]^12^ |
| 6-23 months | $227 | $78-905 |  |
| 2-4 years | $112 | $35-422 |  |
| 5-17 years | $138 | $389-636 |  |
| Hospitalization |  |  | [[24](#_ENREF_24)] |
| 6-23 months, LR | $7,045 | 5815-8275 |  |
| 2 years, LR | $5,703 | 2796-13642 |  |
| 3-4 years, LR | $6,150 | 2460-13866 |  |
| 5-17 years, LR | $7,156 | 447-39920 |  |
| 6-23 months, HR | $9,057 | 2348-24600 |  |
| 2 years, HR | $8,387 | 3019-38466 |  |
| 3-4 years, HR | $11,406 | 3019-38466 |  |
| 5-17 years, HR | $9,617 | 2460-57811 |  |
| Long-term sequelae following influenza-related hospitalization^13^ | $790,567 | $0-1,265,802 | [[25](#_ENREF_25)] |

^8^ Definitions and follow-up for incidence of fever following vaccination vary by study. Rates are 2X higher for higher-risk subgroups.

^9^ Vary by age, calculated by costing out recommended dose of acetaminophen for average weight in each age group.

^10^ Only a proportion of children with influenza illness are assumed to make a physician visit. ICD-9 codes: 487 and 487.0.

^11^ 1993-1997 Marketscan database, The Medstat Group, Ann Arbor, MI.

^12^ 2001-2003 Marketscan database, The Medstat Group, Ann Arbor, MI.

^13^ Includes costs of lifetime care and special education.

| Variable | Most Likely Estimate | Range for Sensitivity Analysis | Source | |
| --- | --- | --- | --- | --- |
| Costs of vaccination |  |  |  | |
| Vaccine dose | $8.60 |  | HHS/BARDA^14^ | |
| Administration |  |  |  | |
| Mass vaccination | $11.30 |  | [[26](#_ENREF_26)], assumption | |
| Physician office setting, existing visit | $13.71 |  | [[27](#_ENREF_27)] | |
| Physician office setting, additional visit | $20.92 |  | [[27](#_ENREF_27)] | |
| Parent time^15^ (hours), mass vaccination setting, 6 m – 4 y | 0.7 | - | [[26](#_ENREF_26)], assumption | |
| Parent time^16^ (hours), physician office setting | 1.0 | - | Assumption | |
| Hourly wage rate for parent time | $20.62 |  | [[28](#_ENREF_28)] | |
| Vaccination-related adverse events |  |  | [[29](#_ENREF_29)] |  |
| Physician visit for injection site reaction^17^ | $77 | $38-864 |  |  |
| Anaphylaxis^18^ | $3,415 | $66-17,404 |  |  |
| Guillain Barré syndrome^19^ | $29,557 | $8,431-99,852 |  |  |
| Quality Adjustments^20,21^ (Disutility associated with an event) |  |  |  |  |
| Episode of influenza | 0.005 | 0.002-0.009 | [[30](#_ENREF_30)] |  |
| Otitis media | 0.042 | 0.023-0.065 | [[31](#_ENREF_31)] |  |
| Non-hospitalized complications (pneumonia) | 0.046 | 0.027-0.071 | [[31](#_ENREF_31)] |  |
| Hospitalization, pneumonia | 0.076 | 0.054-0.100 | [[31](#_ENREF_31)] |  |
| Anaphylaxis | 0.02 | 0.006-0.041 | [[30](#_ENREF_30)] |  |
| Guillain Barré syndrome | 0.141 | 0.092-0.199 | [[30](#_ENREF_30)] |  |

^14^ 2009 government purchase price per dose of inactivated vaccine.

^15^ Includes travel, waiting, and vaccination time.

^16^ Assumes that a proportion of patients who receive their vaccination at the physician’s office will receive vaccinations as an adjunct to an existing visit which incurs 10 minutes in additional recipient time. 66% of vaccinations for recipients ≥65 years, 47% of vaccinations for recipients 50-65 years, and 44% of vaccinations for recipients 18-49 years occurred at an existing visit. Time required for a vaccine-initiated visit is assumed to be 1 hour.

^17^ 5- minute visit, CPT code 99211

^18^ ICD-9 codes: 999.4, 995.0, 995.6x

^19^ ICD-9 code: 357.0

^20^ Quality adjustments are included in the model as a one-time decrement in utility for each temporary health state. For example, an episode of influenza results in a one-time loss of 0.005 quality-adjusted life years [QALYs]. Utility losses were calculated by dividing the discounted time-traded off by the respondent’s discounted life expectancy. In these studies, respondents were asked how much of their own lifetime they would be willing to trade in order to avoid a case of uncomplicated influenza (i.e., a time-tradeoff valuation) and respondents were instructed to include lost time from productive activities in the valuation task.

^21^ Average life span used to calculate total QALYs lost due to life-long sequelae and death was 77.9-78.2 years depending on child’s current age.

References:

1. Monto AS, Sullivan KM (1993) Acute respiratory illness in the community: frequency of illness and the agents involved. Epidemiology and Infection 110: 145-160.

2. Neuzil KM, Mellen BG, Wright PF, Mitchel EF, Griffin MR (2000) The effect of influenza on hospitalizations, outpatient visits, and courses of antibiotics in children. New England Journal of Medicine 342: 225-231.

3. Neuzil KM, Wright PF, Mitchel EF, Griffin MR (2000) The burden of influenza illness in children with asthma and other chronic medical conditions. J Pediatr 137: 856-864.

4. Belshe RB, Mendelman PM, Treanor J, King J, Gruber WC, et al. (1998) The efficacy of live attenuated, cold-adapted, trivalent, intranasal influenzavirus vaccine in children. New England Journal of Medicine 338: 1405-1412.

5. Heikkinen T, Ruuskanen O, Waris M, Ziegler T, Arola M, et al. (1991) Influenza vaccination in the prevention of acute otitis media in children. AJDC 145: 445-448.

6. Heikkinen T, Thint M, Chonmaitree T (1999) Prevalence of various respiratory viruses in the middle ear during acute otitis media. New England Journal of Medicine 340: 260-264.

7. Neuzil KM, Dupont WD, Wright PF, Edwards KM (2001) Efficacy of inactivated and cold-adapted vaccines against inlfuenza A infection, 1985 to 1990: the pediatric experience. Pediatr Infect Dis J 20: 733-740.

8. Belshe RB, Gruber WC, Mendelman PM, Cho I, Reisinger K, et al. (2000) Efficacy of vaccination with live attenuated, cold-adapted, trivalent, intranasal influenza virus vaccine against a variant (A/Sydney) not contained in the vaccine. J Pediatr 136: 168-175.

9. Retailliau HF, Curtis AC, Storr G, Caesar G, Eddins DL, et al. (1980) Ilness after influenza vaccination reported through a nationwide surveillance system, 1976-1977. American Journal of Epidemiology 111: 270-278.

10. Breman JG, Hayner NS (1984) Guillain-Barre syndrome and its relationship to swine influenza vaccination in Michigan, 1976-1977. Am J Epidemiol 119: 880-889.

11. Hurwitz ES, Schonberger LB, Nelson DB, Holman RC (1981) Guillain-Barre syndrome and the 1978-1979 influenza vaccine. N Engl J Med 304: 1557-1561.

12. Langmuir AD, Bregman DJ, Kurland LT, Nathanson N, Victor M (1984) An epidemiologic and clinical evaluation of Guillain-Barre syndrome reported in association with the administration of swine influenza vaccines. Am J Epidemiol 119: 841-879.

13. Marks JS, Halpin TJ (1980) Guillain-Barre syndrome in recipients of A/New Jersey influenza vaccine. Jama 243: 2490-2494.

14. Parkin WE, Beecham HJ, Streiff E, Sharrar RG, Harris JC (1978) Relationship studied in Pennsylvania. Guillain-Barre syndrome and influenza immunization. Pa Med 81: 47-48, 50-42.

15. Rohrer JW, Hamilton GG (1978) Surveillance of the swine influenza vaccination program at the Royal Military College, Kingston. Can Med Assoc J 118: 1528-1530.

16. Roscelli JD, Bass JW, Pang L (1991) Guillain-Barre syndrome and influenza vaccination in the US Army, 1980-1988. Am J Epidemiol 133: 952-955.

17. Safranek TJ, Lawrence DN, Kurland LT, Culver DH, Wiederholt WC, et al. (1991) Reassessment of the association between Guillain-Barre syndrome and receipt of swine influenza vaccine in 1976-1977: results of a two-state study. Expert Neurology Group. Am J Epidemiol 133: 940-951.

18. Schonberger LB, Bregman DJ, Sullivan-Bolyai JZ, Keenlyside RA, Ziegler DW, et al. (1979) Guillain-Barre syndrome following vaccination in the National Influenza Immunization Program, United States, 1976--1977. Am J Epidemiol 110: 105-123.

19. WHO staff-based on an epidemiological report of the Health Department of the Netherlands (1979) Vaccination and Guillain-Barre syndrome. Weekly Epidemiologic Record March: 82-83.

20. Finkelstein JA (2004).

21. Finkelstein JA, Stille CS, Nordin J, Davis R, Raebel MA, et al. (2003) Reduction in antibiotic use among US children, 1996-2000. Pediatrics 112: 620-627.

22. Reference PDRPD (2000) 2000 Redbook: Pharmacy's Fundamental Reference (Red Book Drug Topics). Montvale, N.J.: Thomson PDR.

23. Prosser LA, Bridges CB, Uyeki TM, Hinrichsen VL, Meltzer MI, et al. (2006) Health benefits, risks, and cost-effectiveness of influenza vaccination of children. Emerging Infectious Diseases 12: 1548-1558.

24. Keren R, Zaoutis TE, Saddlemire S, Luan XQ, Coffin SE (2006) Direct medical cost of influenza-related hospitalizations in children. Pediatrics 118 1321-1327.

25. Waitzman NJ, Scheffler RM, Romano PS (1984) The cost of birth defects: Estimates of the value of prevention. Lanham: University Press of America, Inc. 1-262 p.

26. Prosser L, O'Brien M, Molinari N, Hohman K, Nichol K, et al. (2008) Non-traditional settings for influenza vaccination of adults: Costs and cost-effectiveness. Pharmacoeconomics 26: 163-178.

27. Centers for Medicare and Medicaid Services (CMS) (2009) Physician fee schedule.

28. Bureau of Labor Statistics (2009) National Compensation Survey - Mean hourly earnings and weekly hours for selected worker and establishment characteristics Dec 2007-Jan 2009.

29. Molinari NA, Ortega-Sanchez IR, Messonnier ML, Thompson WW, Wortley PM, et al. (2007) The annual impact of seasonal influenza in the US: Measuring disease burden and costs. Vaccine 25: 5086-5096.

30. Prosser LA, Bridges CB, Uyeki TM, Rego VH, Ray GT, et al. (2005) Values for preventing influenza-related morbidity and vaccine adverse events in children. Health and Quality of Life Outcomes 3: 1-16.

31. Prosser LA, Ray GT, O'Brien M, Kleinman K, Santoli J, et al. (2004) Preferences and willingness to pay for health states prevented by pneumococcal conjugate vaccine. Pediatrics 113: 283-290.
