# Supplementary material for: Cost-Effectiveness of 2009 Pandemic Influenza A(H1N1) Vaccination in the United States
Source: PLoS One. 2011 Jul 29;6(7):e22308. doi: 10.1371/journal.pone.0022308 (PMC3146485; doi:10.1371/journal.pone.0022308)
Supplement: Table S2 — Additional model input parameters, 18 years and older. (DOCX) [file pone.0022308.s002.docx]

Table S2. Additional model input parameters, 18 years and older.

| Variable | **Base Case**  **Estimate** | **Range for**  **Sensitivity Analysis** | **Source** |
| --- | --- | --- | --- |
| Probability that adult with influenza makes an outpatient visit |  |  |  |
| 18-64 y, healthy | 0.31 | 0.27 -0.356 | [[1](#_ENREF_1),[2](#_ENREF_2),[3](#_ENREF_3)] |
| 18-64 y, high risk | 2X healthy | 1X-3X | Expert Panel |
| ≥65 years, all | 2X healthy 18-64y | 1X-3X | Expert Panel |
| Probability of vaccine-related adverse events |  |  |  |
| Local reaction, IIV (not medically-attended) | 0.44 | 0.030-0.585 | [[1](#_ENREF_1),[4](#_ENREF_4),[5](#_ENREF_5),[6](#_ENREF_6),[7](#_ENREF_7)], Expert panel |
| Systemic reaction, IIV | 0.011 | 0.00-0.044 | [[4](#_ENREF_4),[5](#_ENREF_5),[7](#_ENREF_7),[8](#_ENREF_8),[9](#_ENREF_9)], Expert panel |
| Anaphylaxis | 0.000001 | 0-0.000002 | [[10](#_ENREF_10)] |
| Guillain Barré syndrome^1^ (medically attended within 6 weeks of vaccination) | 0.000001 | 0 - 0.000115 | [[11](#_ENREF_11),[12](#_ENREF_12),[13](#_ENREF_13),[14](#_ENREF_14),[15](#_ENREF_15),[16](#_ENREF_16),[17](#_ENREF_17),[18](#_ENREF_18),[19](#_ENREF_19),[20](#_ENREF_20)] |
| Influenza-related medical costs |  |  |  |
| OTC medications | $4 |  | [[21](#_ENREF_21)] |
| Outpatient visit, medical costs |  |  |  |
| 18-49 y, healthy | $158 | $128-133 | [[21](#_ENREF_21)] |
| 18-49 y, high risk | $919 | $248-1853 | [[21](#_ENREF_21)] |
| 50-64 y, healthy | $191 | $150-163 | [[21](#_ENREF_21)] |
| 50-64 y, high risk | $928 | $448-1296 | [[21](#_ENREF_21)] |
| ≥65 y, all | $312 | $301-340 | [[21](#_ENREF_21)] |
| Influenza- related hospitalization, medical costs |  |  |  |
| 18-49 y, healthy | $24,080 | $21,300-27,374 | [[21](#_ENREF_21)] |
| 18-49 y, high risk | $60,442 | $43,784-84,985 | [[21](#_ENREF_21)] |
| 50-64 y, healthy | $28,249 | $25,336-31,741 | [[21](#_ENREF_21)] |
| 50-64 y, high risk | $52,319 | $45,891-60,727 | [[21](#_ENREF_21)] |
| ≥65 y, all | $18,103 | $17,310-19,042 | [[21](#_ENREF_21)] |

^1^ Guillain Barré syndrome is modeled over a one-year time horizon including a short-term hospitalization followed by gradual recovery.

| Variable | **Base Case**  **Estimate** | **Range for**  **Sensitivity Analysis** | **Source** |
| --- | --- | --- | --- |
| Vaccination-related adverse events |  |  |  |
| Physician visit for systemic reaction^2^ |  |  | Same as for ILI visit |
| Anaphylaxis^3^  18-49y  50-64y  ≥65 y | $524  $563  $557 | $477-579  $488-652  $411-754 | [[21](#_ENREF_21)], Marketscan database^4^ |
| Guillain-Barré syndrome^5^ | $76,327 | $51,313-102,967 | [[21](#_ENREF_21)], Marketscan database |
| Vaccine dose | $8.60 |  | HHS/BARDA |
| Administration |  |  |  |
| Mass vaccination | $11.30 |  | Assumption |
| Physician office setting, existing visit | $13.71 |  | [[22](#_ENREF_22)] |
| Physician office setting, additional visit | $20.92 |  | [[22](#_ENREF_22)] |
| Recipient time costs^6^ (hours), mass vaccination setting | 0.7 | - | [[23](#_ENREF_23)], assumption |
| Recipient time costs^6^ (hours), physician office setting | 1.0 | - | [[24](#_ENREF_24)] |
| Hourly wage rate | $20.62 |  | [[25](#_ENREF_25)] |

^2^ 5- minute visit, CPT code 99211

^3^ ICD-9 codes: 999.4, 995.0, 995.6x

^4^ 2001-2003 Marketscan database, The Medstat Group, Ann Arbor, MI.

^5^ ICD-9 code: 357.0

^6^ Includes travel, waiting, and vaccination time.

References:

1. Bridges CB, Thompson WW, Meltzer MI, Reeve GR, Talamonti WJ, et al. (2000) Effectiveness and cost-benefit of influenza vaccination of healthy working adults: a randomized controlled trial. JAMA 284 1655-1663.

2. Nichol KL, Mendelman P, Mallon KP, Jackson LA, Gorse GJ, et al. (1999) Effectiveness of live, attenuated intranasal influenza virus vaccine in healthy, working adults: a randomized controlled trial. JAMA 282: 137-144.

3. Nichol KL, Lind A, Margolis KL, Murdoch M, McFadden R, et al. (1995) The effectiveness of vaccination against influenza in healthy, working adults. New England Journal of Medicine 333 889-893.

4. Edwards KM, Dupont WD, Westrich MK, Plummer WD, Jr., Palmer PS, et al. (1994) A randomized controlled trial of cold-adapted and inactivated vaccines for the prevention of Influenza A disease. J Infect Dis 169: 68-76.

5. Nichol KL, Margolis KL, Lind A, Murdoch M, McFadden R, et al. (1996) Side effects associated with influenza vaccination in healthy working adults. A randomized, placebo-controlled trial. Arch Intern Med 156: 1546-1550.

6. Halperin SA, Smith B, Mabrouok T, Germain M, Trepanier P, et al. (2002) Safety and immunogenicity of a trivalent, inactivated, mammalian cell culture-derived influenza vaccine in healthy adults, seniors, and children. Vaccine 20 1240-1247.

7. Scheifele DW, Bjornson G, Johnston J (1990) Evaluation of adverse events after influenza vaccination in hospital personnel. Canadian Medical Association Journal 142 127-130.

8. Treanor JJ, Kotloff K, Betts RF, Belshe R, Newman F, et al. (1999) Evaluation of trivalent, live, cold-adapted (CAIV-T) and inactivated (TIV) influenza vaccines in prevention of virus infection and illness following challenge of adults with wild-type influenza A (H1N1), A (H3N2), and B viruses. Vaccine 18: 899-906.

9. Margolis KL, Nichol KL, Poland GA, Pluhar RE (1990) Frequency of adverse reactions to influenza vaccine in the elderly. A randomized, placebo-controlled trial. [erratum appears in JAMA . 1991;265(21):2810.]. JAMA 264: 1139-1141.

10. Retailliau HF, Curtis AC, Storr G, Caesar G, Eddins DL, et al. (1980) Ilness after influenza vaccination reported through a nationwide surveillance system, 1976-1977. American Journal of Epidemiology 111: 270-278.

11. Breman JG, Hayner NS (1984) Guillain-Barre syndrome and its relationship to swine influenza vaccination in Michigan, 1976-1977. Am J Epidemiol 119: 880-889.

12. Hurwitz ES, Schonberger LB, Nelson DB, Holman RC (1981) Guillain-Barre syndrome and the 1978-1979 influenza vaccine. N Engl J Med 304: 1557-1561.

13. Langmuir AD, Bregman DJ, Kurland LT, Nathanson N, Victor M (1984) An epidemiologic and clinical evaluation of Guillain-Barre syndrome reported in association with the administration of swine influenza vaccines. Am J Epidemiol 119: 841-879.

14. Marks JS, Halpin TJ (1980) Guillain-Barre syndrome in recipients of A/New Jersey influenza vaccine. Jama 243: 2490-2494.

15. Parkin WE, Beecham HJ, Streiff E, Sharrar RG, Harris JC (1978) Relationship studied in Pennsylvania. Guillain-Barre syndrome and influenza immunization. Pa Med 81: 47-48, 50-42.

16. Rohrer JW, Hamilton GG (1978) Surveillance of the swine influenza vaccination program at the Royal Military College, Kingston. Can Med Assoc J 118: 1528-1530.

17. Roscelli JD, Bass JW, Pang L (1991) Guillain-Barre syndrome and influenza vaccination in the US Army, 1980-1988. Am J Epidemiol 133: 952-955.

18. Safranek TJ, Lawrence DN, Kurland LT, Culver DH, Wiederholt WC, et al. (1991) Reassessment of the association between Guillain-Barre syndrome and receipt of swine influenza vaccine in 1976-1977: results of a two-state study. Expert Neurology Group. Am J Epidemiol 133: 940-951.

19. Schonberger LB, Bregman DJ, Sullivan-Bolyai JZ, Keenlyside RA, Ziegler DW, et al. (1979) Guillain-Barre syndrome following vaccination in the National Influenza Immunization Program, United States, 1976--1977. Am J Epidemiol 110: 105-123.

20. WHO staff-based on an epidemiological report of the Health Department of the Netherlands (1979) Vaccination and Guillain-Barre syndrome. Weekly Epidemiologic Record March: 82-83.

21. Molinari NA, Ortega-Sanchez IR, Messonnier ML, Thompson WW, Wortley PM, et al. (2007) The annual impact of seasonal influenza in the US: Measuring disease burden and costs. Vaccine 25: 5086-5096.

22. Centers for Medicare and Medicaid Services (CMS) (2009) Physician fee schedule.

23. Prosser L, O'Brien M, Molinari N, Hohman K, Nichol K, et al. (2008) Non-traditional settings for influenza vaccination of adults: Costs and cost-effectiveness. Pharmacoeconomics 26: 163-178.

24. Coleman MS, Fontanesi J, Meltzer MI, Shefer A, Fishbein DB, et al. (2005) Estimating medical practice expenses from administering adult influenza vaccinations. Vaccine 23 915-923.

25. Bureau of Labor Statistics (2009) National Compensation Survey - Mean hourly earnings and weekly hours for selected worker and establishment characteristics Dec 2007-Jan 2009.
