# Supplementary material for: Cost-Effectiveness of 2009 Pandemic Influenza A(H1N1) Vaccination in the United States
Source: PLoS One. 2011 Jul 29;6(7):e22308. doi: 10.1371/journal.pone.0022308 (PMC3146485; doi:10.1371/journal.pone.0022308)
Supplement: Table S3 — Cumulative cases by week for hypothetical 16-week epidemic. (DOCX) [file pone.0022308.s003.docx]

Table S3. Cumulative cases by week for hypothetical 16-week epidemic

| **Week** | **Cumulative % of**  **Total Cases** |
| --- | --- |
| 1 | 0.01% |
| 2 | 0.03% |
| 3 | 0.10% |
| 4 | 0.35% |
| 5 | 1.14% |
| 6 | 3.66% |
| 7 | 11.13% |
| 8 | 29.21% |
| 9 | 57.61% |
| 10 | 81.75% |
| 11 | 93.67% |
| 12 | 98.00% |
| 13 | 99.40% |
| 14 | 99.83% |
| 15 | 99.97% |
| 16 | 100.00% |
