# Supplementary material for: Cost-Effectiveness of 2009 Pandemic Influenza A(H1N1) Vaccination in the United States
Source: PLoS One. 2011 Jul 29;6(7):e22308. doi: 10.1371/journal.pone.0022308 (PMC3146485; doi:10.1371/journal.pone.0022308)
Supplement: Table S4 — Sensitivity analyses with varying vaccination cost assumptions, $/QALY. (DOCX) [file pone.0022308.s004.docx]

Table S4. Sensitivity analyses with varying vaccination cost assumptions, $/QALY.

a. One vs. two doses for children.^1^

|  | **# Doses Required** | | |
| --- | --- | --- | --- |
| **Age Group** | **2 doses** | **1 dose** | % change |
| 6-23 months, LR | $13,638 | $1,565 | 89% |
| 2 y, LR | $15,954 | $2,543 | 84% |
| 3-4 y, LR | $22,079 | $4,910 | 78% |
| 5-11 y, LR | $20,383 | $5,778 | 72% |
| 12-17 y, LR | $25,307 | $7,953 | 69% |
| 6-23 months, HR | Cost-saving | Cost-saving | - |
| 2 y, HR | Cost-saving | Cost-saving | - |
| 3-4 y, HR | Cost-saving | Cost-saving | - |
| 5-11 y, HR | Cost-saving | Cost-saving | - |
| 12-17 y, HR | Cost-saving | Cost-saving | - |

1. Higher vaccination cost scenario assuming all require an extra visit in the physician office setting.^2^

|  | **Vaccination Costs** | | |
| --- | --- | --- | --- |
| **Age Group** | Physician office setting | Physician office setting |  |
|  | Some vaccination occurs at existing visits | All vaccination requires extra visit | % change |
| 6-23 months, LR | $13,638 | $15,314 | 12% |
| 2 y, LR | $15,954 | $17,814 | 12% |
| 3-4 y, LR | $22,079 | $24,462 | 11% |
| 5-11 y^3^, LR | $34,109 | $37,811 | 11% |
| 12-17 y, LR | $18,989 | $23,386 | 23% |
| 18-49 y, LR | $11,511 | $15,118 | 31% |
| 50-64 y, LR | $38,130 | $48,149 | 26% |
| ≥65 y, all | $75,709 | $109,028 | 44% |
| 6-23 months, HR | Cost-saving | Cost-saving | - |
| 2 y, HR | Cost-saving | Cost-saving | - |
| 3-4 y, HR | Cost-saving | Cost-saving | - |
| 5-11 y^3^, HR | Cost-saving | Cost-saving | - |
| 12-17 y, HR | Cost-saving | Cost-saving | - |
| 18-49 y, HR | Cost-saving | Cost-saving | - |
| 50-64 y, HR | Cost-saving | Cost-saving | - |

^1^ Assuming 15% attack rate and mid-range costs of vaccination.

^2^ 15% influenza illness attack rate

^3^ Assumes children 9 years or younger require 2 doses and children 10-11 years require one dose.

c. Higher cost for vaccine dose (twice as high as in primary analysis), $/QALY.

|  | **Probability of Influenza-Related Hospitalization** | | |
| --- | --- | --- | --- |
| **Age Group** | Assumption from primary analysis | Higher cost for vaccine dose | % change |
| 6-23 months, LR | $13,638 | $17,780 | 30% |
| 2 y, LR | $15,954 | $20,554 | 29% |
| 3-4 y, LR | $22,079 | $27,969 | 27% |
| 5-11 y, LR | $16,207 | $22,744 | 40% |
| 12-17 y, LR | $7,953 | $13,388 | 68% |
| 18-49 y, LR | $8,502 | $12,559 | 48% |
| 50-64 y, LR | $30,960 | $41,392 | 34% |
| ≥65 y, all | $66,011 | $87,662 | 33% |
| 6-23 months, HR | Cost-saving | Cost-saving | - |
| 2 y, HR | Cost-saving | Cost-saving | - |
| 3-4 y, HR | Cost-saving | Cost-saving | - |
| 5-11 y, HR | Cost-saving | Cost-saving | - |
| 12-17 y, HR | Cost-saving | Cost-saving | - |
| 18-49 y, HR | Cost-saving | Cost-saving | - |
| 50-64 y, HR | Cost-saving | Cost-saving | - |

d. Higher cost for vaccine administration costs (25% higher than in primary analysis), $/QALY.

|  | **Probability of Influenza-Related Hospitalization** | | |
| --- | --- | --- | --- |
| **Age Group** | Assumption from primary analysis | Higher vaccine administration costs | % change |
| 6-23 months, LR | $13,638 | $16,049 | 18% |
| 2 y, LR | $15,954 | $18,631 | 17% |
| 3-4 y, LR | $22,079 | $25,507 | 16% |
| 5-11 y, LR | $16,207 | $17,158 | 6% |
| 12-17 y, LR | $7,953 | $8,708 | 9% |
| 18-49 y, LR | $8,502 | $9,548 | 12% |
| 50-64 y, LR | $30,960 | $34,149 | 10% |
| ≥65 y, all | $66,011 | $71,097 | 8% |
| 6-23 months, HR | Cost-saving | Cost-saving | - |
| 2 y, HR | Cost-saving | Cost-saving | - |
| 3-4 y, HR | Cost-saving | Cost-saving | - |
| 5-11 y, HR | Cost-saving | Cost-saving | - |
| 12-17 y, HR | Cost-saving | Cost-saving | - |
| 18-49 y, HR | Cost-saving | Cost-saving | - |
| 50-64 y, HR | Cost-saving | Cost-saving | - |
